# Effects of Salinity on Growth, Muscle Biochemical Components and Antioxidant Resistance of *Planiliza haematocheilus*

**DOI:** 10.3390/ani16203315

**Published:** 2026-10-21

**Authors:** Yilin Wang, Xin’an Wang, Zhifeng Liu, Jingkun Yang, Pengfei Yan, Aijun Ma

**Affiliations:** 1 State Key Laboratory of Mariculture Biobreeding and Sustainable Goods, Yellow Sea Fisheries Research Institute, Chinese Academy of Fishery Sciences, Qingdao 266071, China; 2 College of Fisheries and Life Science, Shanghai Ocean University, Shanghai 201306, China; 3 China-ASEAN Belt and Road Joint Laboratory on Mariculture Technology (Qingdao), Qingdao 266071, China

**Keywords:** mulle, *Planiliza haematocheilus*, salinity, growth, antioxidant

## Abstract

This study evaluated the effects of different water salinities (0, 10, 20 [control], 30, and 40; Practical Salinity Scale, dimensionless) over 56 days on growth performance, muscle biochemical composition, and antioxidant responses in serum and liver of *Planiliza haematocheilus*. After one-week pre-acclimation at 20, juveniles were directly transferred to the experimental salinities; thus, all groups except the 20 control experienced acute salinity transfer followed by 56-day exposure. Therefore, acute transfer stress and subsequent salinity exposure were confounded and could not be separated in this design. Survival was highest at 20 and lowest at 0. Weight gain rate and specific growth rate peaked at 30 and were lowest at 0. Under this direct-transfer protocol, the optimal salinity range for growth and survival was approximately 20–30 for these ~93 g juveniles. Hepatosomatic index differed significantly among salinity groups. Muscle moisture content was negatively correlated with salinity. Crude protein content was highest in the salinity-40 group, whereas crude fat was highest at 20 and lowest at 0; ash content did not differ significantly among treatments. Superoxide dismutase (SOD) activity in serum and liver showed no clear trend with salinity, but most groups differed significantly from the control. Liver SOD activity was highest at 10 and lowest at 30. Serum catalase (CAT) activity did not differ significantly among salinity groups, whereas liver CAT activity tended to decrease and then increase with rising salinity. Salinity had limited effect on serum malondialdehyde (MDA) content, except for a peak at 40, whereas liver MDA content decreased with increasing salinity and was significantly higher in the low-salinity groups. These results indicate that, under the direct-transfer protocol, *P. haematocheilus* juveniles (initial weight ~93 g) pre-acclimated to 20 and then directly transferred showed optimal growth and survival at 20–30, and that liver antioxidant parameters may serve as sensitive biomarkers of salinity stress. However, because acute transfer stress was confounded with subsequent 56-day salinity exposure, these findings are specific to this direct-transfer protocol and developmental stage and should not be extrapolated to gradual acclimation protocols or smaller juveniles.

## Simple Summary

Salinity stress, whether prolonged or acute, profoundly affects respiratory metabolism, growth, development, and immune defense in fish. In severe cases, such stress can induce physiological dysfunction, thereby compromising immune competence and disease resistance. Consequently, elucidating the effects of salinity on fish physiological mechanisms holds critical theoretical and practical value, particularly for optimizing species composition in cage aquaculture and enhancing production efficiency. To systematically investigate the regulatory mechanisms underlying salinity stress responses in mullet, this study analyzed the effects of varying salinities on growth performance, muscle nutritional composition, and tissue antioxidant capacity. The objective was to clarify the physiological acclimation patterns of mullet in fluctuating saline environments, thereby providing a scientific basis for healthy aquaculture management and effective salinity regulation.

## 1. Introduction

Salinity, as an important abiotic factor, primarily influences the physiological state and functions of fish through osmoregulation [1]. Studies have shown that when fish are in water environments close to or slightly above the iso-osmotic point, their osmoregulatory activity remains at a relatively low level, accompanied by reduced energy expenditure [2]. When salinity deviates significantly from the iso-osmotic point, fish are required to consume additional energy to maintain internal osmotic homeostasis [3]. Prolonged or acute salinity stress can further affect respiratory metabolism, growth and development, and immune defense functions [4,5]. In natural water bodies and extensive culture systems such as artificial ponds, water salinity is susceptible to fluctuations due to natural or anthropogenic factors. Such fluctuations can lead to increased oxygen consumption, accelerated metabolism, and elevated energy demands in fish, and in severe cases, may cause physiological dysfunction, thereby impairing immune defense capacity and disease resistance. Ultimately, this hampers normal growth and may even result in mortality [6,7,8].

The effects of salinity on the physiological mechanisms of fish have been comprehensively studied, covering aspects such as embryonic development, growth and survival, tolerance, energy budget, antioxidant systems, and osmoregulation [9,10,11,12]. By exploring the patterns of physiological changes under salinity influence, fish can be reared in suitable salinity environments based on their specific physiological characteristics, thereby improving aquaculture yield and efficiency [8,13]. Particularly for euryhaline fishes that can tolerate low-salinity or freshwater conditions, appropriate salinity acclimation can improve growth performance in low-salinity waters [14]. Therefore, related research holds significant theoretical importance and practical value for optimizing the species structure of marine cage culture and enhancing farming benefits in China.

The mullet (*Planiliza haematocheilus*) is an important traditionally cultured fish species in northern China and a major species among coastal brackish-water aquaculture fish. It is euryhaline and eurythermal, with a salinity tolerance range of 0–38 [15]. Significant progress has been made in research on its morphological characteristics, tissue structure, nutritional requirements, artificial culture techniques, and genetic background [16]. The effects of salinity on the growth and antioxidant performance of mullet have been reported previously. Zhang et al. (2020) conducted a 6-week rearing experiment on juvenile mullet with an initial body weight of (8.78 ± 1.67) g under salinity conditions of 0–25, evaluating the physiological effects of low-salinity stress from the perspectives of growth and antioxidant indices [15]. To further systematically elucidate the regulatory mechanisms of salinity stress on the growth and physiology of mullet, the present study used larger juveniles and a broader salinity range than Zhang et al. (2020) [15] (see Materials and Methods), and analyzed the effects of different salinities on growth performance, muscle nutritional composition, and tissue antioxidant capacity. The present study aims to clarify the physiological acclimation patterns of mullet in changing salinity environments and to provide a theoretical basis for healthy aquaculture and salinity regulation.

## 2. Materials and Methods

### 2.1. Source of Materials

The experimental mullets were obtained from the Jiaozhou base of Qingdao Baorong Aquatic Products Co., Ltd. Qingdao, China, all from the same batch. They had been safely overwintered in a greenhouse, with uniform size, good vitality, and no injuries or diseases. Their average body weight was (92.99 ± 7.25) g, and average body length was (20.58 ± 1.56) cm.

### 2.2. Experimental Method

The experiment established five salinity groups: 0, 10, 20 (control), 30, and 40. Salinities are reported on the Practical Salinity Scale (PSS-78; UNESCO, 1983) and are therefore dimensionless. The numerical values throughout the text, tables, and figures refer to this scale. After one-week acclimation in cement tanks at 20 salinity, fish were fasted for 24 h. Immediately before transfer, all fish (*n* = 300 in total) were individually weighed to the nearest 0.01 g and measured for body length to the nearest 0.1 cm; these values were recorded as the initial body weight and body length. Fish were then directly transferred to 1 m^3^ cylindrical plastic tanks pre-adjusted to 0, 10, 20, 30, and 40 without gradual salinity adjustment. Thus, all groups except the 20 control experienced an acute salinity change at transfer, followed by 56 days of exposure; the 20 group served as the non-transfer control. Each treatment had three replicates, with 20 fish per replicate, totaling 300 fish. The experimental water was prepared as follows: high-salinity groups were formulated using filtered underground brine water supplemented with artificial sea salt; low-salinity groups were prepared by mixing filtered underground brine water with fully aerated tap water; and the freshwater group (0) used fully aerated tap water directly. The experiment lasted 56 days under natural temperature conditions (18–25 °C). During the rearing period, continuous aeration was provided, and one-third of the water was replaced daily to maintain water quality. Fish were fed to satiation twice daily at 8:00 and 16:00 with floating pellet feed (nutritional composition: crude protein 35.33%, crude fat 4.93%, ash 12.81%).

The experimental unit was the tank, not the individual fish. Each salinity treatment had three independent tanks (*n* = 3). Within each tank, five fish were measured for growth and three fish for liver/antioxidant assays; these individual values were averaged to obtain one tank-level value before statistical analysis. Therefore, all analyses used *n* = 3 tanks per salinity, and no pseudoreplication occurred.

### 2.3. Indicator Measurement and Calculation Methods

Before the beginning and at the end of the experiment, fish were starved for 24 h before sampling. At each sampling time, five fish were randomly selected [from each replicate], and their body weight and body length were measured. The body weight and body length recorded at the beginning and end of the experiment were designated as initial body weight (*W*_0_), initial body length (*L*_0_), final body weight (*W*_1_), and final body length (*L*_1_), respectively. Of these five fish, three were randomly selected, immediately dissected, and their livers were removed and weighed individually using an electronic balance (*W_L_*; accurate to 0.001 g). The experiment lasted 56 days (*t* = 56 d). The initial stocking number per replicate group was *N*_0_, and the number of surviving fish at the end of the experiment was *N*_1_. The measured indicators included survival rate, weight gain rate, specific growth rate, hepatosomatic index, and condition factor, and were calculated as follows:

Survival rate (SR, %) = *N*_1_/*N*_0_ × 100%

Weight gain rate (WG, %) = (*W*_1_ − *W*_0_)/*W*_0_ × 100%

Specific growth rate (SGR, %/d) = (Ln*W*_1_ − Ln*W*_0_)/*t* × 100%

Hepatosomatic index (HIS, %) = (*W_L_*/*W*_1_) × 100%

Condition factor (CF) = *W*_1_/*aL*_1_*^b^*

where *W*_1_ and *L*_1_ are the observed final body weight and body length of the same fish, respectively; *W_L_* is the liver weight of the same fish; *aL*_1_*^b^* is the expected body weight at body length *L*_1_; and *a* and *b* are the regression parameters of the weight–length relationship (*W* = *aL^b^*).

### 2.4. Sample Collection and Measurement

After the experiment, the fish were starved for 24 h, and then 3 individuals were randomly selected from each replicate for sampling. Prior to sampling, the fish were anesthetized with MS-222 (100 mg/L) and placed on an ice plate. Blood was collected from the caudal vein (or caudal artery) using a pre-cooled 1 mL syringe. The blood samples were left to stand at 4 °C overnight, then centrifuged at 3500 r/min for 5 min at low temperature. The supernatant was transferred into pre-cooled 1 mL centrifuge tubes and stored at −20 °C until analysis. After blood collection, the liver and a portion of dorsal muscle were rapidly dissected from the mullets, immediately rinsed with pre-cooled physiological saline, blotted dry with filter paper, placed in sample bags, and stored at −20 °C for further analysis.

For the determination of hepatic antioxidant indices, a measured amount of liver tissue was placed into pre-cooled 1.5 mL centrifuge tubes on ice and minced with scissors. The minced tissue was transferred to a glass homogenizer, and 9 volumes (*v*/*w*) of pre-cooled physiological saline were added for homogenization. The homogenate was centrifuged at 3500 r/min for 10 min at low temperature in a refrigerated centrifuge, and the supernatant was collected for assay.

Superoxide dismutase (SOD) activity was determined by the xanthine oxidase method. One unit (U) of SOD activity was defined as the amount of enzyme required to achieve 50% inhibition of the SOD reaction in the assay system according to the kit instructions. Catalase (CAT) activity was measured by the ultraviolet spectrophotometric method, with one unit defined as the amount of enzyme that decomposes 1 μmol of H_2_O_2_ per second per milliliter of serum or per milligram of protein. Malondialdehyde (MDA) content was determined by the thiobarbituric acid (TBA) method. The antioxidant indices and protein content (by the BCA method) were all measured using kits supplied by Nanjing Jiancheng Bioengineering Institute, Nanjing, China, and all operations were performed in accordance with the corresponding manufacturer’s instructions.

Muscle biochemical composition was analyzed following national standard methods: moisture content was determined by the oven-drying method at 105 °C; crude protein by the Kjeldahl method; crude fat by the Soxhlet extraction method; and crude ash by the muffle furnace incineration method at 550 °C.

### 2.5. Data Processing

All data were analyzed using one-way analysis of variance (ANOVA) with SPSS Statistics for Windows, version 18.0 (SPSS Inc., Chicago, Ill., USA). Group differences were compared using Duncan’s multiple range test, and statistical significance was set at *p* < 0.05.

## 3. Results

### 3.1. Effect of Salinity on the Growth Performance of Mullet

**Figure 1:**
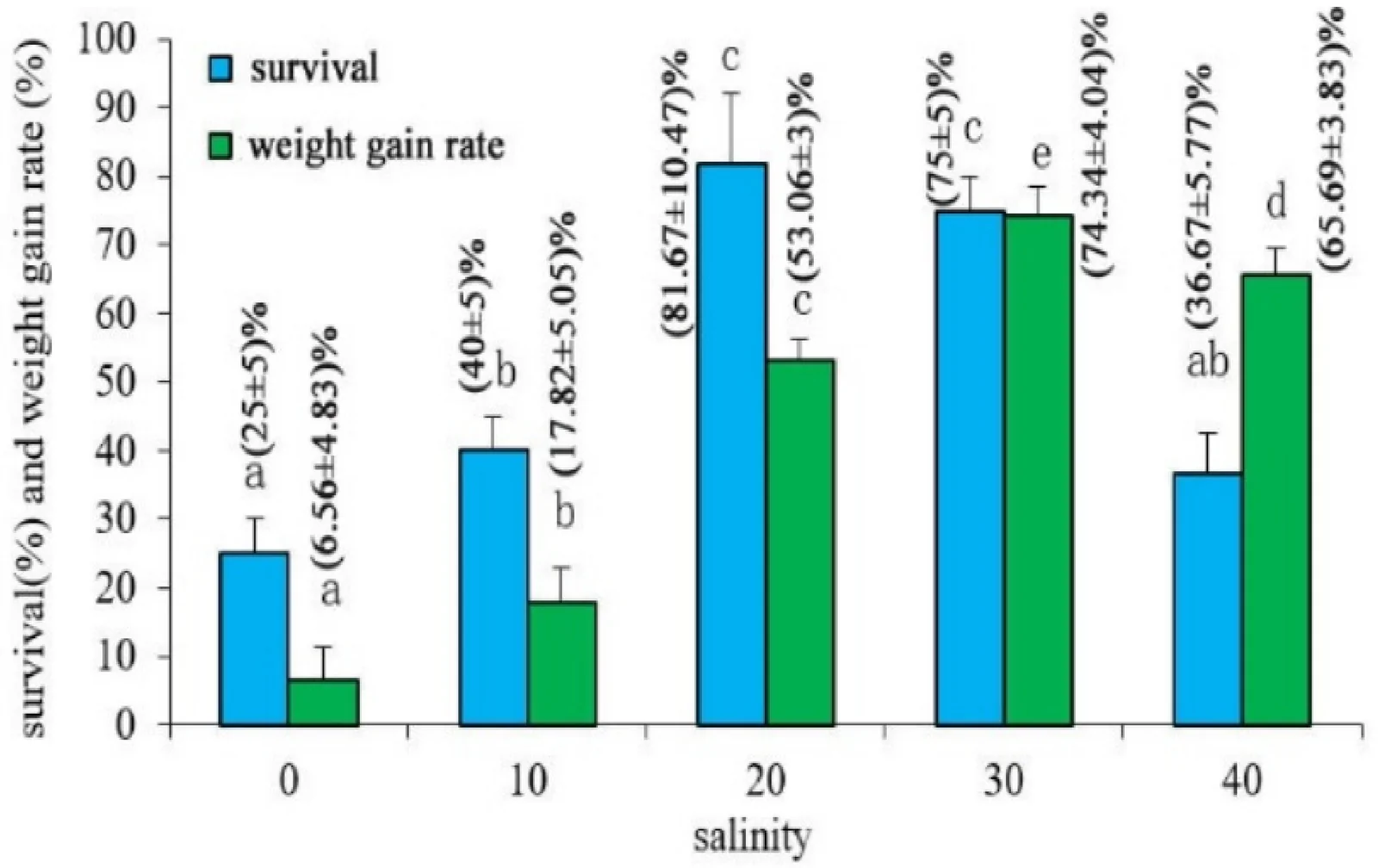
Effects of salinity on survival rate and weight gain rate of mullet. Note: Different letters within the same series in Figure 1 and Figure 2 indicate statistically significant differences (*p* < 0.05).

**Figure 2:**
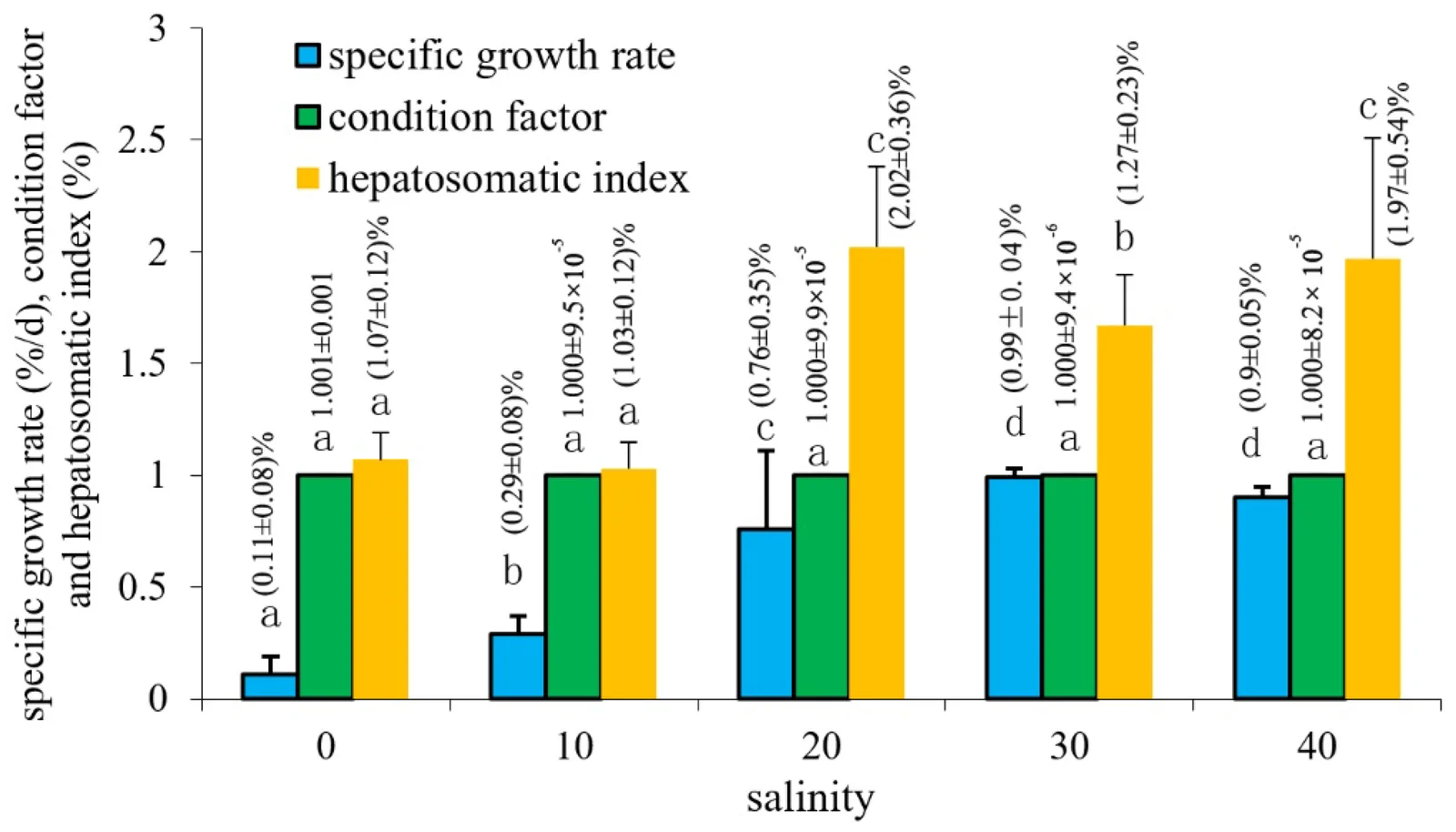
Effects of salinity on specific growth rate, condition factor, and hepatosomatic index of mullet. Note: Different letters within the same series in Figure 1 and Figure 2 indicate statistically significant differences (*p* < 0.05).

One-way ANOVA was performed separately to assess the effects of salinity on each growth performance index of mullet, and the results are presented in Table 1. Among the salinity groups, except for condition factor, which was not significantly affected (*p* = 0.501), all other growth performance indexes showed highly significant differences (*p* < 0.001). Further multiple comparisons were conducted using Tukey HSD’s method, and the results are shown in Figure 1 and Figure 2. The results were as follows: the survival rate was highest in the control group (20) and decreased in all other salinity groups; among them, the freshwater group (0) had the lowest survival rate, at only 25%. Except for the 30 group, which showed no significant difference from the control group (20) (*p* > 0.05), all other groups exhibited significant differences from the control group (*p* < 0.05). The weight gain rate and specific growth rate both showed significant differences among all salinity groups (*p* < 0.05), and both parameters increased first and then decreased with increasing salinity, reaching their maximum values in the 30 group and their minimum values in the freshwater group (0). The condition factor was relatively low in the 30 group; however, there was no significant difference in the condition factor among all salinity groups (*p* > 0.05). The hepatosomatic index reached its highest value in the control group (20), decreased at 30, and increased again at 40.

### 3.2. Effect of Salinity on the Biochemical Composition of Mullet Muscle

**Figure 3:**
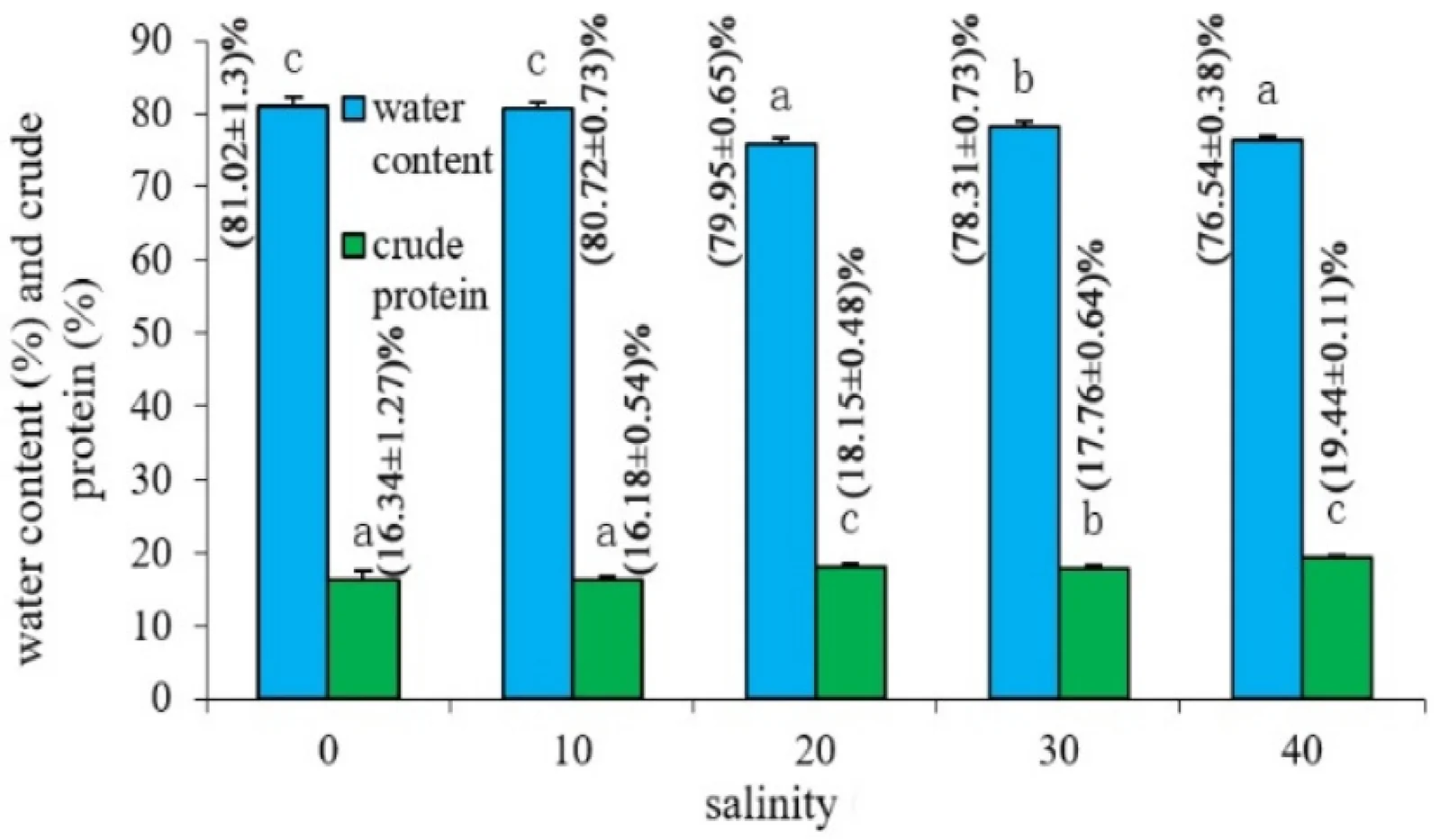
The effect of salinity on the water content and crude protein of mullet. Note: Different letters within the same series in Figure 3 and Figure 4 indicate statistically significant differences (*p* < 0.05).

**Figure 4:**
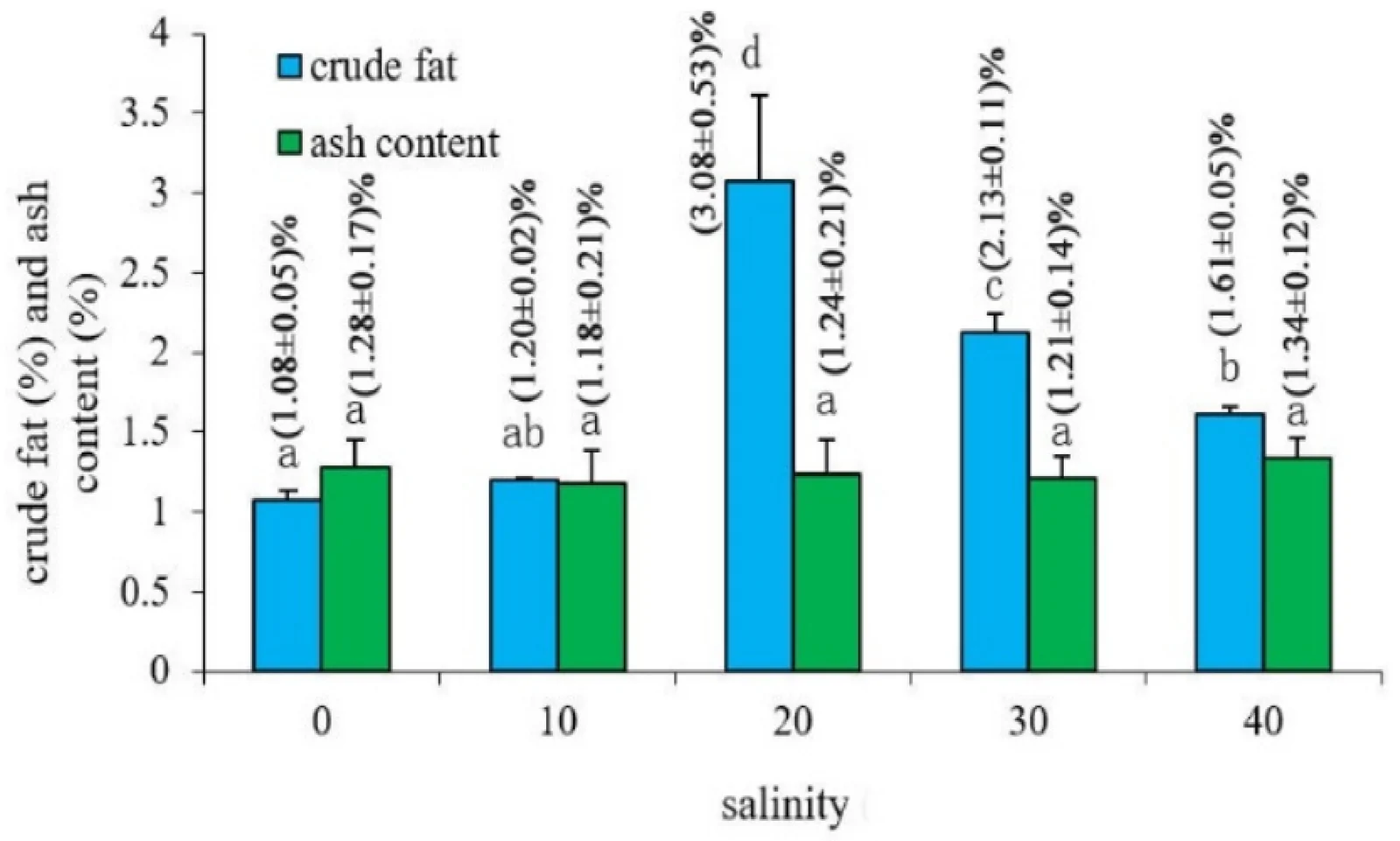
The effect of salinity on crude fat and ash content in mullet. Note: Different letters within the same series in Figure 3 and Figure 4 indicate statistically significant differences (*p* < 0.05).

One-way ANOVA was performed separately to analyze the effects of salinity on the biochemical components of mullet muscle, and the results are shown in Table 2. Among the salinity groups, except for ash content, which was not significantly affected (*p* = 0.815), all other biochemical components showed highly significant differences (*p* < 0.001). Further multiple comparisons were conducted using Tukey HSD’s method, and the results are presented in Figure 3 and Figure 4. Compared with the control group (salinity 20), except for the salinity-40 group, muscle moisture content and crude protein content in all direct-transfer groups (i.e., all groups transferred directly to other salinities) differed significantly from those in the control group (salinity 20) (*p* < 0.05). Muscle moisture content decreased with increasing salinity, showing a negative correlation with salinity. Crude protein content was highest in the salinity-40 group. The crude fat content was highest in the control group (3.08%), and no significant differences were found between the 10 group and the freshwater group (0) or the 40 group (*p* > 0.05), whereas significant differences existed among all other groups (*p* < 0.05); the freshwater group had the lowest crude fat content (1.08%), forming a marked contrast with the control group. In addition, no significant differences in muscle crude ash content were observed among all groups (*p* > 0.05).

### 3.3. Effect of Salinity on the Antioxidant Performance of Mullet

#### 3.3.1. Effects of Salinity on SOD Activity in Mullet

**Figure 5:**
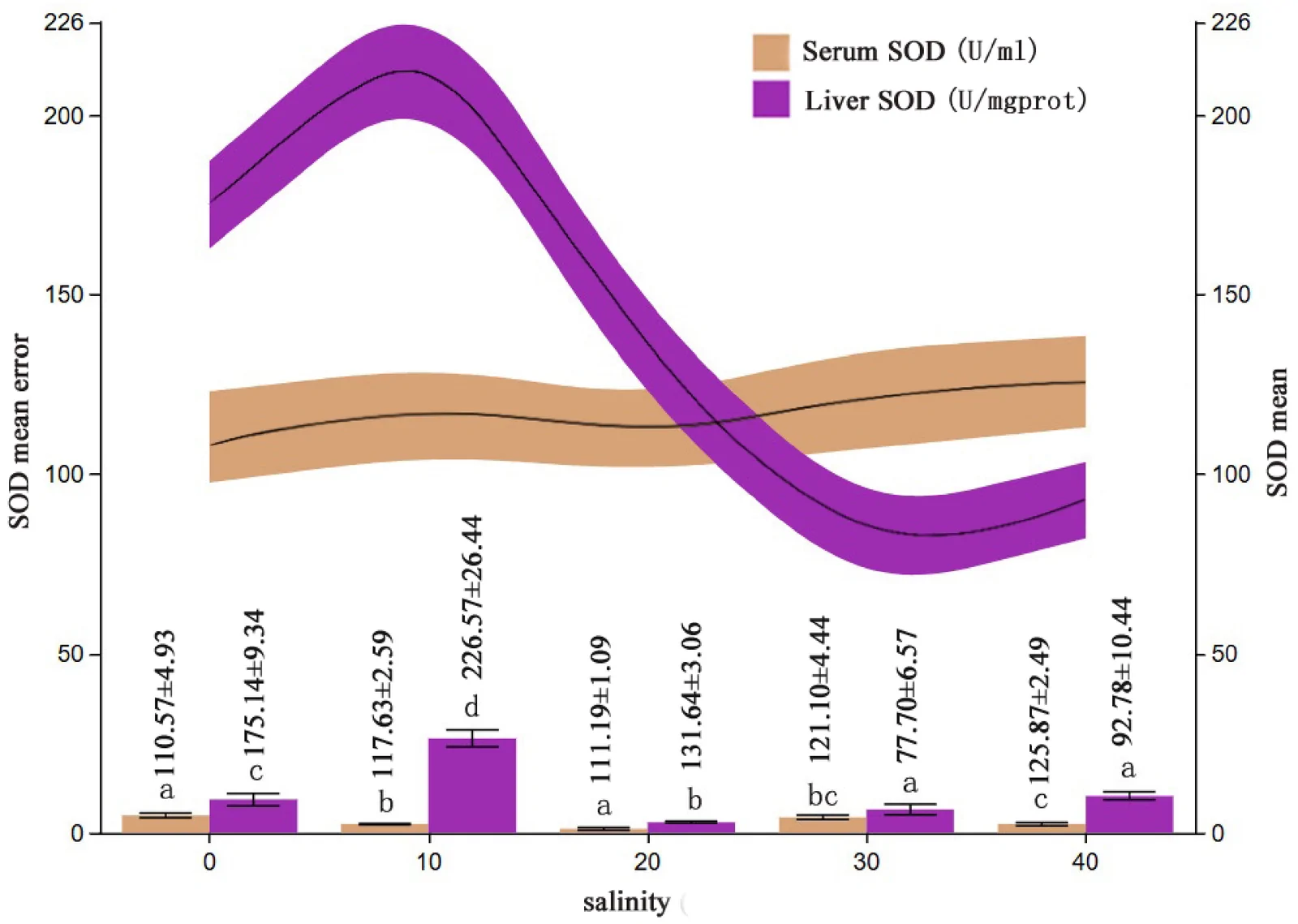
Effects of salinity on SOD activity in serum and liver of mullet. Note: Different letters within the same tissue indicate statistically significant differences (*p* < 0.05).

One-way analysis of variance (ANOVA) was performed separately to examine the effect of salinity on serum and liver SOD activity, and the results are shown in Table 3. Salinity had a significant effect on serum SOD activity (*p* = 0.013) and a highly significant effect on liver SOD activity (*p* = 0.000016). The Tukey HSD method was further used for multiple comparisons, and the results are shown in Figure 5. The effect of salinity on the activities of the two tissues did not follow a consistent pattern. Serum SOD activity showed no significant difference between the freshwater group (0) and the control group (20) (*p* > 0.05), while all other groups differed significantly from the control group (*p* < 0.05). Among them, the lowest value was observed in the 0 group (110.46 U/mL), and the highest value was in the 40 group (125.87 U/mL). The variation in liver SOD activity was markedly greater than that in serum, with the highest value (226.5 U/mgprot) occurring in the 10 group and the lowest value (77.70 U/mgprot) in the 30 group. All inter-group differences were statistically significant (*p* < 0.05).

#### 3.3.2. Effects of Salinity on CAT Activity in Mullet

**Figure 6:**
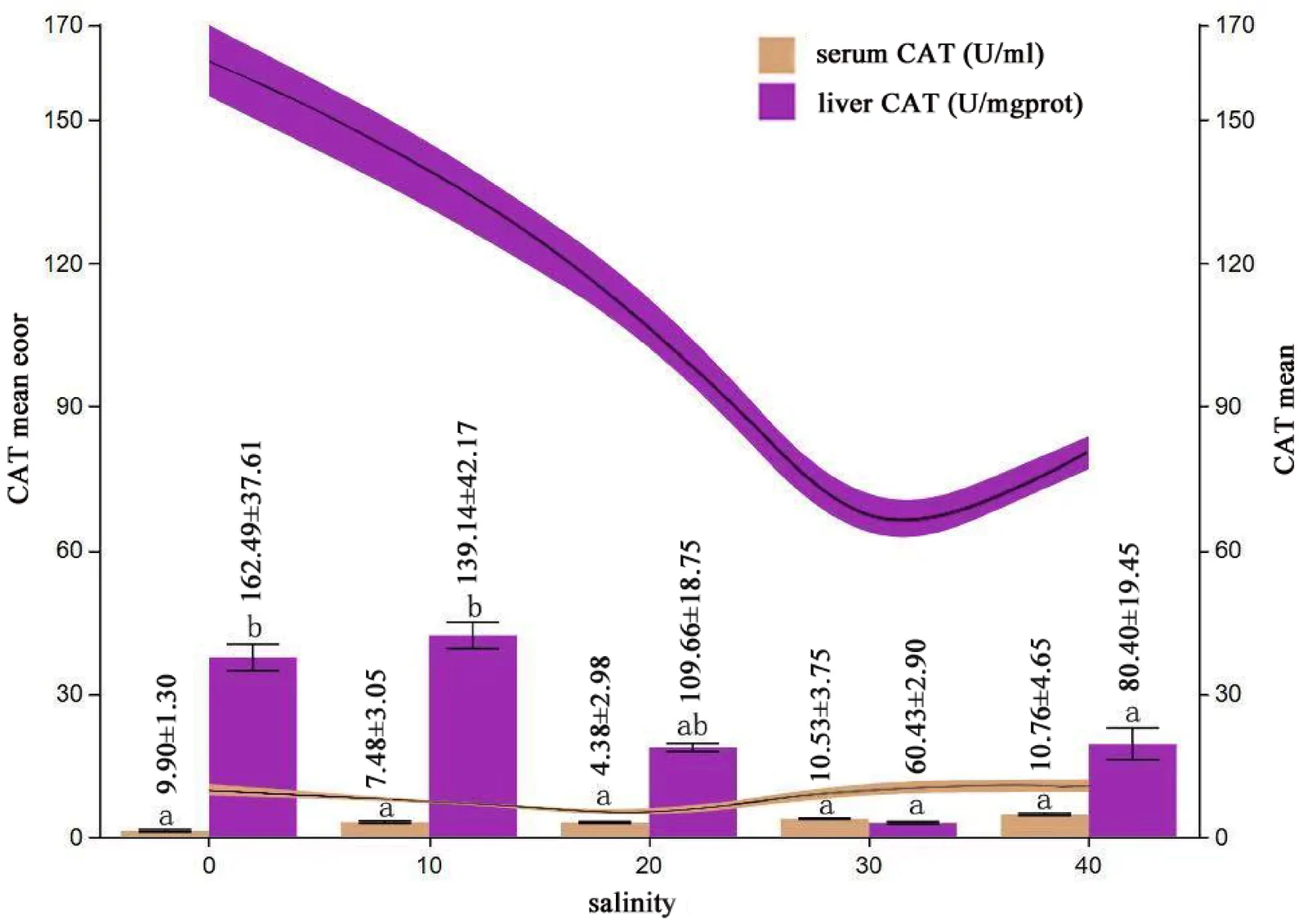
Effects of salinity on CAT activity in serum and liver of mullet. Note: Different letters within the same tissue indicate statistically significant differences (*p* < 0.05).

One-way analysis of variance (ANOVA) was performed separately to examine the effect of salinity on serum and liver CAT activity, and the results are shown in Table 3. Salinity had no significant effect on either serum or liver CAT activity (*p* > 0.05). Tukey HSD multiple comparison test was further used, and the results are shown in Figure 6. The effects of salinity on CAT activities in the serum and liver of mullet are shown: with increasing salinity, both serum and liver CAT activities exhibited a trend of initially decreasing and then increasing. However, the differences in serum CAT activity among salinity treatment groups were not statistically significant (*p* > 0.05). Serum CAT activity reached its lowest value at 20 salinity (the control group), at 4.40 U/ml, whereas liver CAT activity reached its minimum at 30 salinity, at 60.43 U/mg prot.

#### 3.3.3. Effects of Salinity on MDA Content in Mullet

**Figure 7:**
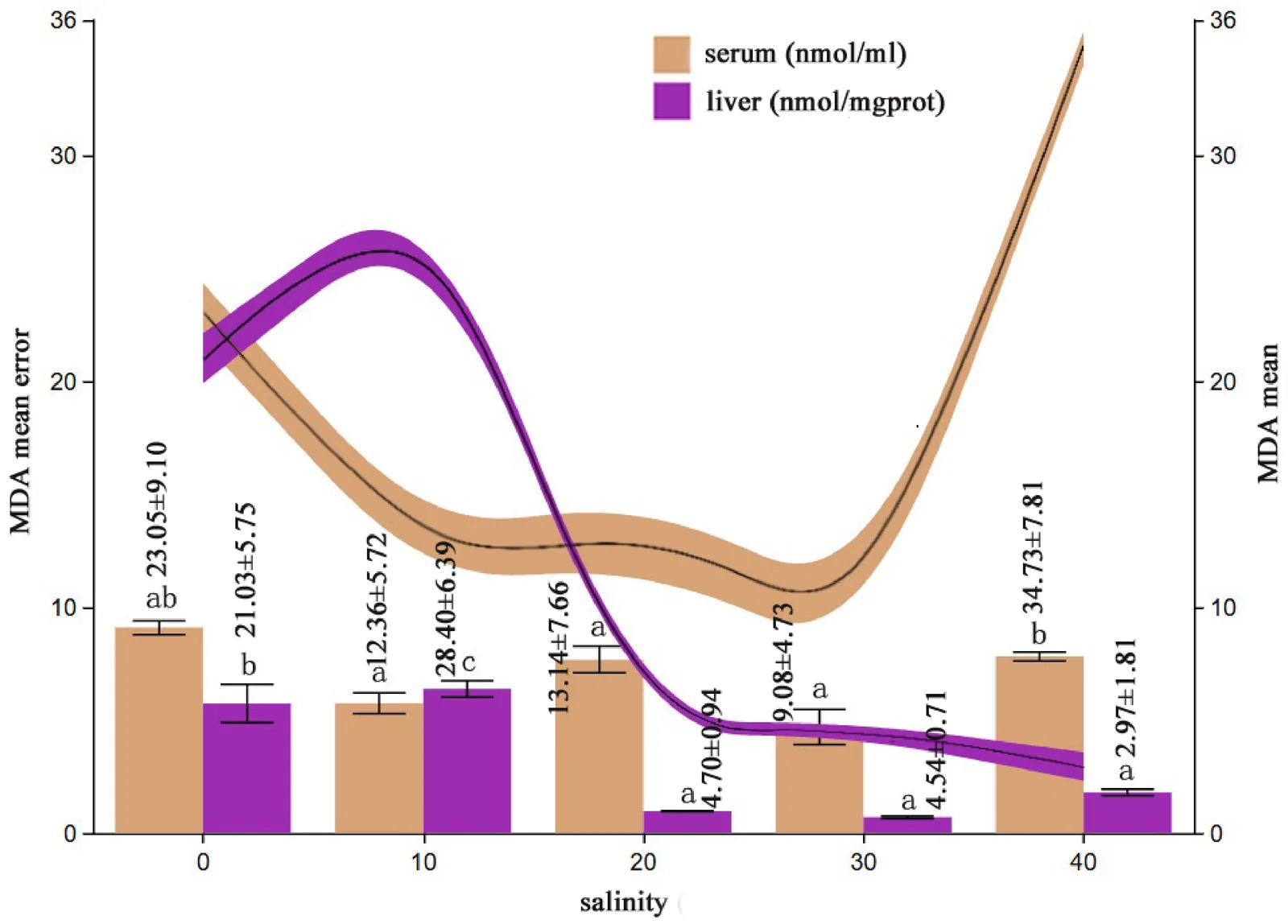
Effects of salinity on MDA content in serum and liver of mullet. Note: Different letters within the same tissue indicate statistically significant differences (*p* < 0.05).

One-way ANOVA was performed separately to examine the effects of salinity on MDA content in the serum and liver of mullet, and the results are shown in Table 3. Salinity had no significant effect on serum MDA content (*p* = 0.062), but had a highly significant effect on liver MDA content (*p* = 0.001). Tukey HSD multiple comparison test was further used for multiple comparisons, and the results are shown in Figure 7. Serum MDA content was less affected by salinity; except for the 40 salinity group, which reached the peak value (34.73 nmol/mL), there were no significant differences among the other groups (*p* > 0.05). Liver MDA content showed a gradual decreasing trend with increasing salinity, reaching the lowest value (2.97 nmol/mgprot) in the highest salinity group, and this group did not differ significantly from the control group (20) (*p* > 0.05). The liver MDA contents in the two low-salinity groups (0 and 10) were significantly higher than that in the control group, and there was also a significant difference between these two groups (*p* < 0.05).

## 4. Discussion

### 4.1. Effects of Salinity on Growth Indicators of Mullet

Salinity is an important environmental factor affecting the survival and reproduction of fish. Changes in external water salinity can impact various physiological activities of fish, including survival, growth, reproduction, digestion and absorption, and immune function, to varying degrees [17]. Studies have shown that different fish species exhibit significant differences in salinity tolerance ranges and adaptive capacities [8,13]. *Epinephelus coioides* grows faster and utilizes feed more efficiently within a salinity range of 12–18, while its immune performance is effectively activated and disease resistance is enhanced [18,19]. Juvenile pearl gentian grouper (*Epinephelus fuscoguttatus* ♀ × *E. lanceolatus* ♂) exhibits promoted growth and improved intestinal digestive capacity at salinities of 20–25 [8]. For turbot (*Scophthalmus maximus*), a salinity of 18 is more conducive to healthy growth [10].

In the present study, the survival rate of juvenile mullet was highest at 20 salinity (control group). The survival rate in the 30 group was slightly lower but not significantly different from the control (*p* > 0.05). The survival rates of the other groups, in descending order, were 10, 40, and freshwater (0). This trend is similar to the findings of Hua et al. (2006), indicating that mullet fingerlings have the strongest adaptability to seawater, and gradual salinity reduction can significantly improve their stocking survival in freshwater environments [20]. The weight gain rate and specific growth rate of mullet showed a trend of first increasing and then decreasing with rising salinity, peaking in the 30 group, with statistically significant differences compared to other salinity groups (*p* < 0.05); in the freshwater group (0), both growth parameters were the lowest. This result is inconsistent with previous reports by Jiang et al. (2005) and Zhuo et al. (2011), who respectively reported good growth and significant economic benefits of mullet in freshwater aquaculture [21,22]. However, the low survival and growth in the 0 group should not be interpreted as evidence that mullet cannot be cultured in freshwater. Previous successful freshwater culture has generally involved gradual desalination and/or earlier developmental stages. In contrast, our fish were overwintered at 20 and directly transferred to freshwater, causing acute hyposmotic stress. Hua et al. (2006) showed that gradual salinity reduction significantly improves freshwater stocking survival [20]. In addition, Zhang et al. (2020) reported 98.98–100% survival at 0–10 for 8.78 g juveniles, whereas our 92.99 g juveniles showed only 25–40% survival under direct transfer [15]. Specifically, for mullet weighing (8.78 ± 1.67) g at salinities of 0 and 10, survival rates were 98.98% and 100%, weight gain rates were 45.79% and 58.13%, and specific growth rates were 0.89%/d and 1.09%/d, respectively [15]; in the present study, for mullet weighing (92.99 ± 7.25) g at the same salinities, the corresponding survival rates were 25% and 40%, weight gain rates were 6.56% and 17.82%, and specific growth rates were 0.11%/d and 0.29%/d. Clearly, the survival and growth performance of the larger mullet (92.99 ± 7.25 g) were much lower than those of the smaller size class (8.78 ± 1.67 g) under the same salinity conditions. This indicates that developmental stage greatly influences the salinity tolerance and growth response of mullet. Therefore, the discrepancy with previous freshwater studies is likely due to differences in acclimation protocol, developmental stage, and experimental conditions, rather than a general unsuitability of freshwater for mullet. This developmental-stage effect is further discussed in Section 4.4.

In the evaluation of fish growth, condition factor reflects body shape and nutrient accumulation, serving as an important indicator of growth status; hepatosomatic index is a key parameter for assessing fish health [23]. Zhang et al. (2012) reported that the hepatosomatic index of mullet significantly increased with increasing dietary lipid levels (*p* < 0.05), and condition factor reached its maximum at a lipid level of 8.23%; integrated analysis determined this lipid level as optimal for mullet growth, at which the hepatosomatic index was 1.86% [24]. In our study, the condition factor of juvenile mullet was lowest in the 30 group and highest in the freshwater group (salinity 0). There was no significant difference in condition factor among the salinity groups (*p* > 0.05), suggesting that salinity did not significantly affect condition factor and that condition factor is not a sensitive indicator. The lowest condition factor in the 30 group may reflect changes in body shape and energy allocation during rapid growth, whereas the highest condition factor in the 0 group is more likely a survivorship bias/selective mortality effect associated with the high mortality (only 25% survival) rather than evidence that freshwater is beneficial for body shape. Regarding the hepatosomatic index, except for the highest value in the control group (20), the other groups showed an increasing trend with rising salinity, with significant differences among all groups (*p* < 0.05). The dietary lipid level used in our experiment was 4.93%, which does not exceed the suitable range for mullet growth, indicating that salinities of 20 and 30 are more conducive to hepatic lipid deposition in mullet.

### 4.2. Effects of Salinity on Biochemical Composition of Muscle in Mullet

The factors affecting fish muscle quality include genetics, nutrition, aquaculture environment, and rearing mode [10]. Among these, salinity not only regulates fish behavior, feeding, and immune status, but also influences physiological metabolic processes, thereby altering the accumulation and distribution of carbohydrates, lipids, and proteins in the body, which directly or indirectly determines the texture, flavor, and nutritional composition of muscle. Studies on grass carp (*Ctenopharyngodon idellus*) and northern snakehead (*Channa argus*) by Li et al. (2007 and 2008) indicated that appropriate salinity could significantly improve the chemical composition and physical properties of muscle, thereby enhancing muscle quality [25,26].

The results of the present study showed that muscle moisture content in mullet was negatively correlated with water salinity. Except for the 40 group, muscle moisture content in all other salinity groups differed significantly from that of the control group (20) (*p* < 0.05), indicating that mullet could effectively regulate body water balance and maintain homeostasis under high-salinity conditions. This finding is similar to that reported for half-smooth tongue sole [27], but differs from reports on orange-spotted grouper [28] and starry flounder [29], where muscle moisture content showed no significant changes (*p* > 0.05) under different salinity conditions. In terms of nutritional components, crude protein content in muscle did not differ significantly between the control group and the salinity-40 group (*p* > 0.05); crude lipid content reached the highest level in the control group (20) and was lower in the low-salinity groups (freshwater and 10 groups). This is consistent with the metabolic feature that under low-salinity stress, flathead mullet (*Mugil cephalus*) uses protein as the main energy source, suggesting that mullet under low-salinity environments need to mobilize more nutrients to maintain osmotic balance. In addition, during the low-salinity rearing period, sudden salinity changes may have led to reduced feed intake in mullet, which in turn prompted the body to break down stored fat to meet physiological demands; this may also be one of the reasons for the decreased crude lipid content. No significant changes (*p* > 0.05) were observed in crude ash content among all groups.

### 4.3. Effects of Salinity on Antioxidant Performance of Mullet

During long-term evolution, fish have developed antioxidant defense mechanisms centered on antioxidant enzymes and non-enzymatic complexes. Studies have shown that changes in the activities of antioxidant enzymes such as SOD and CAT, as well as the levels of lipid peroxidation products like MDA, can serve as key physiological indicators reflecting the response of the fish antioxidant system to external environmental stress [30].

Fish blood parameters are closely related to their metabolic, nutritional, and disease status [31], among which serum antioxidant indicators can effectively reflect physiological or pathological changes in fish and serve as important bases for evaluating their environmental adaptability. Studies have shown that the response patterns of serum antioxidant indicators to salinity stress vary among different fish species. For example, Shi et al. (2013) found that in silver pomfret (*Pampus argenteus*) under 96 h short-term stress, serum catalase (CAT) activity exhibited a trend of initial increase followed by recovery [32]. Wang et al. (2005) reported that in black rockfish (*Sebastes schlegelii*) under chronic stress, both superoxide dismutase (SOD) and CAT activities gradually increased with decreasing salinity [33]. In the present study, after 56 days of long-term rearing, serum SOD activity in mullet returned to a balanced state without showing obvious regular changes, and no significant differences in CAT activity were observed among salinity groups (*p* > 0.05). In addition, MDA content was higher in the 40 and freshwater (0) groups, which may be due to incomplete recovery from stress responses induced by excessive salinity gradients, but this did not affect the normal survival of mullet. Except for the 40 group, MDA content did not differ significantly among the other salinity groups (*p* > 0.05). In summary, salinity had a relatively limited effect on the serum antioxidant indicators of mullet.

In studies on the salinity adaptation of different fish species, the hepatic antioxidant responses exhibit species-specificity. Wang et al. (2011) found that in *Siganus guttatus*, SOD activity in gill tissue was significantly higher in the 5 salinity group than in other salinity groups, while no significant differences were observed among salinity groups in other tissues [34]. CAT activity showed an increasing trend with decreasing salinity across all tissues, but the differences among groups were not significant [34]. Notably, in that study, the SOD and CAT activities in the liver of *S. guttatus* were higher than those in gill, kidney, and muscle tissues, a finding consistent with that of Zhao et al. (2008) in *Acipenser schrenckii* [30]. However, similar trends are not consistently observed across all fish species. Guo et al. (2014) reported that in *Paralichthys olivaceus*, under acute stress on day 6, there were no significant inter-group differences in hepatic SOD and CAT activities or MDA content [35]. Fang et al. (2014) found that in *Cynoglossus semilaevis*, hepatic SOD activity showed significant differences only between the 20 group and the freshwater group, while CAT activity increased with decreasing salinity [36]. Clearly, the response of hepatic antioxidant indices to salinity stress is species-specific, likely related to differences in ecological habits, acclimation strategies, and physiological regulatory mechanisms among species. The results of the present study show that in the liver of mullet, SOD activity exhibits an “S”-shaped trend of first increasing, then decreasing, and then increasing again with rising salinity; CAT activity shows a trend of first decreasing and then increasing; while MDA content initially increases and then decreases with rising salinity. This indicates that under long-term low-salinity stress, fish activate the antioxidant defense system through osmoregulation to cope with oxidative stress caused by environmental changes. The fact that MDA content in the liver of mullet did not show an increasing trend after its initial decrease with rising salinity suggests that even under high-salinity conditions of 40, the fish can maintain a certain physiological homeostasis by mobilizing its own antioxidant system without exceeding its tolerance limit, thereby exhibiting some degree of growth adaptability. Within the salinity range of 0–25, different developmental stages of mullet exhibit differential responses in antioxidant factors. Zhang et al. (2020) showed that in mullet with a body weight of (8.78 ± 1.67) g, SOD and CAT activities and MDA content all exhibited a “first decrease and then increase” trend [15]. However, within the same salinity range, in the present study, mullet with a body weight of (92.99 ± 7.25) g displayed a different pattern: SOD and MDA showed a “first increase and then decrease” trend, while CAT activity continuously decreased [15]. These results indicate that the antioxidant responses of mullet under salinity stress exhibit clear developmental stage-specificity, which may be related to differences in physiological adaptation mechanisms at different growth stages.

### 4.4. Developmental Stage-Dependent Salinity Responses

The present results, together with previous data on *P. haematocheilus*, indicate that salinity responses are strongly developmental-stage dependent. Zhang et al. (2020) reared smaller juveniles (8.78 ± 1.67 g) at 0–25 for 6 weeks and reported survival rates of 98.98% and 100% at 0 and 10, with weight gain rates of 45.79% and 58.13% and specific growth rates of 0.89 and 1.09% d^−1^, respectively [15]. In contrast, the present study used much larger juveniles (92.99 ± 7.25 g) and, under the same nominal salinities of 0 and 10, observed survival rates of only 25% and 40%, weight gain rates of 6.56% and 17.82%, and specific growth rates of 0.11 and 0.29% d^−1^. This striking discrepancy cannot be attributed solely to salinity itself, because the 20 control fish grew and survived well. Instead, it suggests that larger, seawater-acclimated juveniles are less tolerant to acute hyposmotic transfer than smaller juveniles, possibly because of developmental differences in gill, kidney, and intestinal osmoregulatory capacity, metabolic rate, energy allocation, and antioxidant system maturity. Developmental stage may also influence feed intake and digestive performance under osmotic stress. Consistent with this idea, Hua et al. (2006) showed that gradual salinity reduction significantly improves freshwater stocking survival of mullet fingerlings [20], indicating that both developmental stage and acclimation protocol modulate salinity tolerance.

In addition, the antioxidant responses differed between size classes within the comparable salinity range. Smaller juveniles showed a “decrease-then-increase” pattern in SOD, CAT, and MDA, whereas larger juveniles in the present study displayed different response patterns, with liver antioxidant indices being more sensitive to salinity stress than serum indices. These differences indicate that salinity tolerance and oxidative stress responses are not fixed species-specific traits but are modulated by developmental stage, acclimation history, and the rate of salinity change. Therefore, the optimal salinity range of 20–30 observed here should be considered specific to approximately 93 g juveniles acclimated to 20 and directly transferred to the experimental salinities. It should not be extrapolated to smaller juveniles, broodstock, or fish subjected to gradual salinity acclimation. Future studies should compare multiple size classes and include gradual-acclimation controls to disentangle acute transfer effects from long-term adaptive responses.

## 5. Conclusions

This study demonstrates that, under a direct salinity transfer protocol, *P. haematocheilus* juveniles (initial weight ~93 g) pre-acclimated to 20 and then directly transferred to the experimental salinities showed optimal growth and survival at 20–30. Because all groups except the 20 control experienced acute salinity transfer followed by 56-day exposure, the effects of acute transfer stress and long-term salinity exposure were confounded and could not be separated in this design. After the 56-day exposure, mullet were able to regulate antioxidant enzyme activities, which may have contributed to maintaining physiological homeostasis under the tested conditions. Liver antioxidant indices were more responsive to salinity variation than serum indices in this study and may serve as potential biomarkers of salinity stress in this species, but this conclusion is restricted to the present direct-transfer conditions and juvenile size class. These findings are specific to the direct-transfer protocol and developmental stage and should not be extrapolated to gradual acclimation protocols or smaller juveniles. Future studies should include gradual-acclimation controls, multiple size classes, and time-series sampling after transfer to disentangle acute transfer effects from long-term salinity adaptation in mullet.

## Figures and Tables

**Table 1:** ANOVA of growth performance of *P. haematocheilus* at different salinities (*n* = 3 repetitions).

Growth Performance	Source of Variation	Sum of Squares	*df*	Mean Square	*F*	*p*
	Between groups	7550.000	4	1887.500	43.558	<0.001
Survival rate	Within groups	433.333	10	43.333		
	Total	7983.333	14			
	Between groups	10,675.189	4	2668.797	150.255	<0.001
Weight gain rate	Within groups	177.618	10	17.762		
	Total	10,852.807	14			
	Between groups	1.807	4	0.452	128.112	<0.001
Specific growth rate	Within groups	0.035	10	0.004		
	Total	1.842	14			
	Between groups	0.000	4	0.000	0.897	0.501
Condition factor	Within groups	0.000	10	0.000		
	Total	0.000	14			
	Between groups	8.295	4	2.074	48.933	<0.001
Hepatosomatic index	Within groups	1.695	40	0.042		
	Total	9.990	44			

**Table 2:** ANOVA of biochemical composition of *P. haematocheilus* muscle at different salinities (*n* = 3 repetitions).

Biochemical Composition	Source of Variation	Sum of Squares	*df*	Mean Square	*F*	*p*
	Between groups	64.942	4	16.235	24.402	<0.001
Water content	Within groups	6.653	10	0.665		
	Total	71.595	14			
	Between groups	21.952	4	5.488	10.764	0.001
Crude protein	Within groups	5.099	10	0.510		
	Total	27.051	14			
	Between groups	8.000	4	2.000	33.364	<0.001
Crude fat	Within groups	0.599	10	0.060		
	Total	8.600	14			
	Between groups	0.047	4	0.012	0.384	0.815
Ash content	Within groups	0.305	10	0.030		
	Total	0.351	14			

**Table 3:** ANOVA of antioxidant performance of *P. haematocheilus* at different salinities (*n* = 3 repetition).

Antioxidant Performance	Source of Variation	Sum of Squares	*df*	Mean Square	*F*	*p*
	Between groups	512.009	4	128.002	5.51	0.013
Serum SOD activity	Within groups	232.322	10	23.232		
	Total	744.331	14			
	Between groups	87.332	4	21.833	0.981	0.460
Serum CAT	Within groups	222.546	10	22.255		
	Total	309.878	14			
	Between groups	1318.244	4	329.561	3.199	0.062
Serum MDA	Within groups	1030.100	10	103.010		
	Total	2348.344	14			
	Between groups	44,717.343	4	11,179.336	29.488	<0.001
Liver SOD	Within groups	3791.203	10	379.120		
	Total	48,508.546	14			
	Between groups	20,812.394	4	5203.099	3.309	0.057
Liver CAT	Within groups	15,724.608	10	1572.461		
	Total	36,537.003	14			
	Between groups	1621.640	4	405.410	12.916	0.001
Liver MDA	Within groups	313.893	10	31.389		
	Total	1935.532	14			

## Data Availability

The datasets analyzed are available from the corresponding author on reasonable request.
